# Uncovering neural pathways underlying bulimia nervosa: resting-state neural connectivity disruptions correlate with maladaptive eating behaviors

**DOI:** 10.1007/s40519-023-01617-5

**Published:** 2023-10-30

**Authors:** Jia-ni Wang, Miao Wang, Guo-wei Wu, Wei-hua Li, Zi-ling Lv, Qian Chen, Zheng-han Yang, Xiao-hong Li, Zhen-chang Wang, Zhan-jiang Li, Peng Zhang, Li-rong Tang

**Affiliations:** 1 Department of Radiology, Beijing Friendship Hospital, Capital Medical University, No. 95 Yong An Road, Xicheng District, Beijing, China; 2 Chinese Institute for Brain Research, Beijing, China; 3 CAS Key Laboratory of Behavioral Science, Institute of Psychology, Chinese Academy of Sciences, Beijing, China; 4 Beijing Anding Hospital, Capital Medical University, Beijing, China; 5 The National Clinical Research Center for Mental Disorders and Beijing Key Laboratory of Mental Disorders, No. 5 Ankang Hutong, Xicheng District, Beijing, China

**Keywords:** Bulimia nervosa, Surface-based regional homogeneity, Functional connectivity, Principal component analysis, Eating behavior, Visual information

## Abstract

**Purpose:**

Bulimia nervosa (BN) is characterized by recurrent binge-eating episodes and inappropriate compensatory behaviors. This study investigated alterations in resting-state surface-based neural activity in BN patients and explored correlations between brain activity and eating behavior.

**Methods:**

A total of 26 BN patients and 28 healthy controls were enrolled. Indirect measurement of cerebral cortical activity and functional connectivity (FC) analyses were performed in Surfstat. A principal component analysis (PCA) model was used to capture the commonalities within the behavioral questionnaires from the BN group.

**Results:**

Compared with the healthy control group, the BN group showed decreased surface-based two-dimensional regional homogeneity in the right superior parietal lobule (SPL). Additionally, the BN group showed decreased FC between the right SPL and the bilateral lingual gyrus and increased FC between the right SPL and the left caudate nucleus and right putamen. In the FC–behavior association analysis, the second principal component (PC2) was negatively correlated with FC between the right SPL and the left caudate nucleus. The third principal component (PC3) was negatively correlated with FC between the right SPL and the left lingual gyrus and positively correlated with FC between the right SPL and the right lingual gyrus.

**Conclusion:**

We revealed that the right SPL undergoes reorganization with respect to specific brain regions at the whole-brain level in BN. In addition, our results suggest a correlation between brain reorganization and maladaptive eating behavior. These findings may provide useful information to better understand the neural mechanisms of BN.

**Level of evidence:**

V, descriptive study.

**Supplementary Information:**

The online version contains supplementary material available at 10.1007/s40519-023-01617-5.

## Introduction

Bulimia nervosa (BN) is a distinct eating disorder that features recurrent binge eating episodes and inappropriate compensatory behaviors to prevent weight gain [1, 2]. The reason it is important for clinicians to recognize bulimia is that patients can be at risk for mortality, morbidity and associated psychiatric comorbidities [3]. Cognitive-behavioral therapy is the most commonly used treatment method for BN, but there are a considerable number of patients who achieve only moderate therapeutic effects from this treatment [4]. The unclear neurobiological basis of BN has greatly hindered the development of therapies for this disorder.

In recent years, major advances in neuroimaging in the field of neuroscience, including improvements in functional magnetic resonance imaging (fMRI), have increased the knowledge of the interrelationship between neural mechanisms and BN. For instance, task-based fMRI indicated extensive hyperactivation of the parieto-occipital cortex during alerting in patients with BN compared to healthy controls (HCs), allowing researchers to investigate alterations in the neural patterns that underlie attention dysfunction in BN [5]. Uher et al. found that patients with BN showed greater occipital activity than HCs when viewing food images, and they also found that patients with BN in general demonstrated weaker activation of the occipitotemporal cortex and parietal cortex than HCs in response to body image stimuli [6, 7]. Similarly, Skunde et al. [8] found reduced activation in the right dorsal striatum (caudate nucleus and putamen) in participants with BN compared to HCs when they were confronted with visual food stimuli by asking them to choose eight favorite food images. Furthermore, Wang et al. found disturbed resting-state functional connectivity (FC) within visuospatial networks mediating body image perception and distinct FC changes across subcortical striatal subregions (lentiform nucleus, putamen, caudate, and pallidum) [9]. Another resting-state fMRI study has shown that people with BN have reduced resting-state FC in the somatosensory cortex, which may be involved in body image processing and play an important role in the maintenance and development of BN symptoms [10]. These alterations in functional neural activity may be involved in the clinical manifestations of BN, such as episodic binge eating, self-regulatory control defects, and distorted body image perception.

Regional homogeneity (ReHo) is frequently chosen to analyze changes in brain function changes in research studies [11, 12]; ReHo assumes coherence in spontaneous neural activity between a given voxel and the neighboring voxels in a voxelwise analysis [13]. However, the volume-based three-dimensional ReHo (3dReHo) approach ignores interparticipant variability in gyrus size and shape, which may introduce inaccuracies in the anatomical positioning of functional data, and voxels near the boundary between the gray matter and white matter (WM) exhibit significant local volume effects [14, 15]. The surface-based two-dimensional ReHo (2dReHo) approach focuses on neural activation in the cerebral cortex, significantly decreases signal contamination between neighboring functional brain regions, and improves the test–retest reliability of activity data originating from the cortex [14]. Surface-based 2dReHo was developed to reduce interparticipant variability and to increase statistical power, revealing the intrinsic functional organization of the cortex more clearly than whole-brain volume.

FC is defined as the temporal dependence of neuronal activity patterns, which indicates the level of coactivation between functional time series of anatomically separated brain regions and is considered to reflect functional connections between brain regions at the whole-brain level, including cortical and subcortical structures [16]. Hence, FC is inferred by measuring the correlation between time-series parameters of neuronal activity in different locations [16, 17].

Although there have been studies exploring alterations in brain function in BN patients, there is currently a lack of research specifically investigating resting-state cortical activity in individuals with BN. Based on previous neuroimaging studies indicating alterations in brain functional activity in patients with BN, we used a novel surface-based 2dReHo approach and seed-based FC to explore the differences in brain functional activity between BN patients and HCs in the present study. We hypothesized, first, that patients with BN would have alterations in the functional activity of the cerebral cortex including areas that support reward, self-regulation, somatosensory and visuospatial processing, etc. Second, these regions of the cerebral cortex with abnormal activity would show a reorganization of FC patterns with cortical and/or subcortical structures at the whole-brain level. Finally, changes in brain cortical function activity in patients would correlate with the maladaptive eating behaviors that are common among people with BN.

## Methods

### Participants

A total of 36 patients with BN (2 males) and 32 HCs (2 males) were initially enrolled in this study. All participants were of Han Chinese ethnicity. The patients with BN were recruited from the inpatient and outpatient departments of the hospital. HCs were recruited via the internet. The diagnosis of BN was made by a psychiatrist with specialized knowledge of eating disorders via Mini-International Neuropsychiatric Interview (MINI) 7.0.2 [18] for the Diagnostic and Statistical Manual of Mental Disorders, Fifth Edition (DSM-5) [2], and it is a structured interview. To avoid potential confounding, we excluded patients who currently had other major psychiatric disorders, such as schizophrenia, bipolar disorder, or dissociative disorder, according to MINI; please refer to Table S1 for details. Twelve participants in the BN group had had anorexia nervosa (AN) in their lifetime, but they did not meet the DSM-5 criteria for AN in the 15 months prior to the study. Some participants in the BN group had comorbid anxiety and/or depression at the time of the study; please refer to Table S1 for details. Nineteen patients with BN used to take lorazepam, estazolam or SSRIs for a short period of time, but all of them had stopped medication two months prior to the MRI examination. HCs were required to be within a normal weight range and to be free of any history of psychiatric illness. To rule out any potential psychiatric illness, we conducted a professional face-to-face interview with each HC using MINI 7.0.2 for DSM-5. Further exclusion criteria for all participants were a history of brain injury with a loss of consciousness, a history of neurological disorders, pervasive developmental disorder, intellectual disability, alcohol/substance dependence, smoking, pregnancy, claustrophobia, and any implant constituting a contraindication to MRI. Of the 36 patients enrolled, three patients had metal implants, and one had claustrophobia; according to the exclusion criteria, these four patients did not undergo an MRI examination.

On the day of the MRI examination, all participants were asked to come to the hospital at 8 a.m. after an overnight fast and to undergo blood biochemistry tests to exclude metabolic diseases such as hyperthyroidism, hyperlipidemia and hyperglycemia. We recorded the frequency of binge eating/purging episodes per week during the past month and the duration of illness in patients with BN. In the same visit, all participants were asked to complete the following scale assessments: a visual analog scale [19] (VAS, to assess hunger and satiety before the MRI examination); the Dutch Eating Behavior Questionnaire (DEBQ), which measures three eating behaviors (emotional eating, external eating and restrained eating) [20]; the 26-item Eating Attitude Test (EAT) [21]; the bulimia subscales of the Eating Disorder Inventory-1 (EDI-1) [22]; the 20-item Self-rating Anxiety Scale (SAS) [23]; and the 21-item Beck Depression Inventory (BDI) [24]. Both reliability and the validity of the above scales have been verified in the Chinese population [20–24].

### Data acquisition

A 3.0-T MRI system (Prisma, Siemens, Erlangen, Germany) and a 64-channel phased array coil were used to acquire MRI data. Before all the sequences, a conventional axial T2-weighted image of the brain was acquired to exclude participants with any obvious brain abnormalities. Functional images were obtained using a multislice gradient-echo echo-planar imaging (EPI) sequence with the following parameters: 240 time points; 33 sections with 3.5-mm thickness; repetition time (TR) = 2000 ms; echo time (TE) = 30 ms; flip angle = 90°; matrix = 64 × 64 and field of view (FOV) = 224 × 224 mm^2^. Sagittal structural images spanning the whole brain and were acquired using a 3D magnetization-prepared rapid-acquisition gradient-echo (3D-MPRAGE) sequence with the following parameters: 192 sections with 1-mm thickness; TR/TE = 2530/2.98 ms; inversion time (TI) = 1100 ms; flip angle = 7°; matrix = 256 × 256 and FOV = 256 × 256 mm^2^. The scanning time for each patient was 14 min.

Participants lay supine in the scanner with foam pads to minimize possible micromovements of the head, and they wore earplugs attenuate noise from the scanner. Prior to the examination, participants were asked to close their eyes, stay awake, and breathe quietly during the scan. After the examination, we routinely asked the participants whether they slept during the scan, and they all said no.

### Data preprocessing

fMRIprep [25] and eXtensible Connectivity Pipelines (XCP_D) [26] tools were used to preprocess the resting-state fMRI data. The steps for preprocessing data were as follows: (1) a deformation field correction was applied by using fMRIPrep’s “fieldmap-less” approach; (2) the BOLD reference map was coregistered to the high-resolution T1-weighted (T1w) reference image by using bbregister (FreeSurfer); (3) the images were realigned to correct for head motion and extract motion parameters by using mcflirt; (4) 3dTshift was used for slice-timing correction; (5) the T1w map was processed by the FreeSurfer “recon all” procedure to reconstruct surface maps for participants; (6) grayordinate files containing 91 k samples were generated for each participant by using wb_command; (7) the BOLD time series were resampled onto the 64 k surface map; (8) intensity outliers in each vertex’s time series were interpolated using AFNI’s 3dDespike utility, and the data were further processed by demeaning as well as removal of any linear or quadratic trends; (9) 36 nuisance regressors (including motion parameters, WM signals, cerebrospinal fluid (CSF) signals and global signals) were regressed from resting-state BOLD data; (10) a bandpass filter of 0.01–0.08 Hz was used to filter the residual time series. For the details of preprocessing, please refer to the supplementary material.

In the head-motion correction step, data from 6 patients and 4 HCs were excluded based on the exclusion criterion (> 2.0° of head rotation or > 2.0 mm displacement; mean framewise displacement (FD) ≥ 0.15 mm). The number of excluded volumes was few for each subject, and there was no difference between the two groups (*t* = − 0.88, *p* = 0.38). The number of excluded volumes for the HCs group was 2.16 ± 3.79, and for the BN group, it was 2.96 ± 4.33. Ultimately, 26 patients (1 male) and 28 HCs (2 males), for a total of 54 participants, were included for further analysis.

### Surface-based 2dReho calculation

For each hemisphere in the 64k grayordinate surface file, ReHo was computed using surface-based 2dReHo [27]. Specifically, for each vertex on the surface, the nearest neighbor vertices were identified, and Kendall’s coefficient of concordance (KCC) was calculated to yield ReHo.

### Seed-based FC analysis

The areas that showed significant differences between HCs and patients in surface-based 2dReho were used to perform seed-based FC analysis at the whole-brain level [28]. For each cluster, we extracted the mean time series and calculated the Pearson correlation coefficient with each vertex on the surface and each voxel on the subcortex. Then, Fisher’s Z-transform was applied to the R value in preparation for further statistical analysis.

### Statistical analysis

Statistical analysis was performed using SPSS version 26.0 and R (4.1.3). Demographic and clinical data were compared between patients with BN and HCs using independent t tests for continuous variables and Chi-square tests for categorical variables. Two-sample *t* tests were performed to identify the group differences in surface-based 2dReHo by using Surfstat [29]. The age, educational level and mean FD of each participant were taken as covariates to avoid any undetected effects. Furthermore, two-sample *t* tests were conducted to determine the differences in all possible connections between the participants with BN and the HCs, once again controlling for age, educational level and mean FD. All results mentioned above for the surface were corrected at the statistical threshold of *P* < 0.05 by using the clusterwise random field theory (RFT) method [30] implemented in Surfstat [29]. In addition, the comparison of subcortical FC was corrected by applying a false discovery rate (FDR) correction at the voxel level.

A principal component analysis (PCA) model was used to capture the commonalities within the behavioral questionnaires of the BN group. As a dimensionality-reduction method, PCA is useful for studying multiple correlated phenotypes. All 9 variables were included in the PCA (VAS, the frequency of binge eating/purging per week, DEBQ-Emotional, DEBQ-Externality, DEBQ-Restraint, EDI-BN, EAT, BDI, and SAS). PCA components that had a high ratio of variance and an eigenvalue greater than 1 were retained [31]. These PCA components were further used to perform FC-behavior association analysis. Furthermore, a multiple variable linear regression model (lm function in *R*) was further built to predict the PCA component by FC features to test the association between behaviors and FC features in the BN group. The backward solution was used to determine the best model by using the step function in the stats package, and the false discovery rate (FDR) correction was used to control the rate of false-positives caused by multiple comparisons.

## Results

### Demographic data

Demographic data are shown in Table 1. Patients with BN and HCs were well matched for sex, age, body mass index (BMI) and education. Furthermore, the FD values; the number of binge-eating/purging episodes per week; the duration of illness; and the DEBQ, EDI-BN, EAT, BDI and SAS scores are included in Table 1. No significant differences were found in the FD values between patients with BN and HCs. There were significant differences in DEBQ, EDI-BN, EAT, BDI and SAS scores between patients with BN and HCs.

**Table 1:** Demographic and clinical data of participants

Variables	Bulimia nervosa patients	Healthy controls	Statistics	
	(*n* = 26)		*t*	*p*
Sex (female/male)	(25/1)	(26/2)		> 0.99^a^
Age (y)	24.38 ± 4.96	25.29 ± 2.46	− 0.854	0.397^b^
FD	0.095 ± 0.03	0.097 ± 0.03	− 0.234	0.816^b^
BMI (kg/m^2^)	20.20 ± 2.60	20.71 ± 1.85	− 0.844	0.402^b^
Education (y)	16.50 ± 2.06	16.93 ± 1.92	− 0.79	0.433^b^
VAS	5.19 ± 1.23	4.93 ± 1.88	0.603	0.549^b^
Frequency	6.08 ± 4.29			
Duration of illness (m)	19.75 ± 14.14			
DEBQ-Restraint	37.23 ± 6.81	27.32 ± 8.10	4.878	< 0.001^b,^*
DEBQ-Emotional	47.50 ± 11.08	26.68 ± 11.42	6.801	< 0.001^b,^*
DEBQ-Externality	34.23 ± 6.21	30.54 ± 3.98	2.583	0.013^b,^*
EDI-BN	34.54 ± 5.50	1.32 ± 2.09	28.912	< 0.001^b,^*
EAT	41.58 ± 11.26	11.57 ± 9.69	10.461	< 0.001^b^*
BDI	23.00 ± 10.91	4.67 ± 6.47	7.44	< 0.001^b,^*
SAS	54.38 ± 12.79	33.66 ± 8.35	6.992	< 0.001^b,^*

Data are presented as the mean ± standard deviation, *p < 0.05

*y* years, *m* months, *FD* framewise displacement, *BMI* body mass index, *VAS* Visual Analog Scale, *Frequency* the number of binge-eating/purging episodes per week, *DEBQ* Dutch Eating Behavior Questionnaire, *EDI* Eating Disorders Inventory, *EAT* Eating Attitude Test, *BDI* Beck Depression Inventory, *SAS* Self-Anxiety Scale

^a^Chi-squared test

^b^Two-sample *t* tests

### Surface-based 2dReHo changes in patients with BN versus HCs

**Fig. 1:**
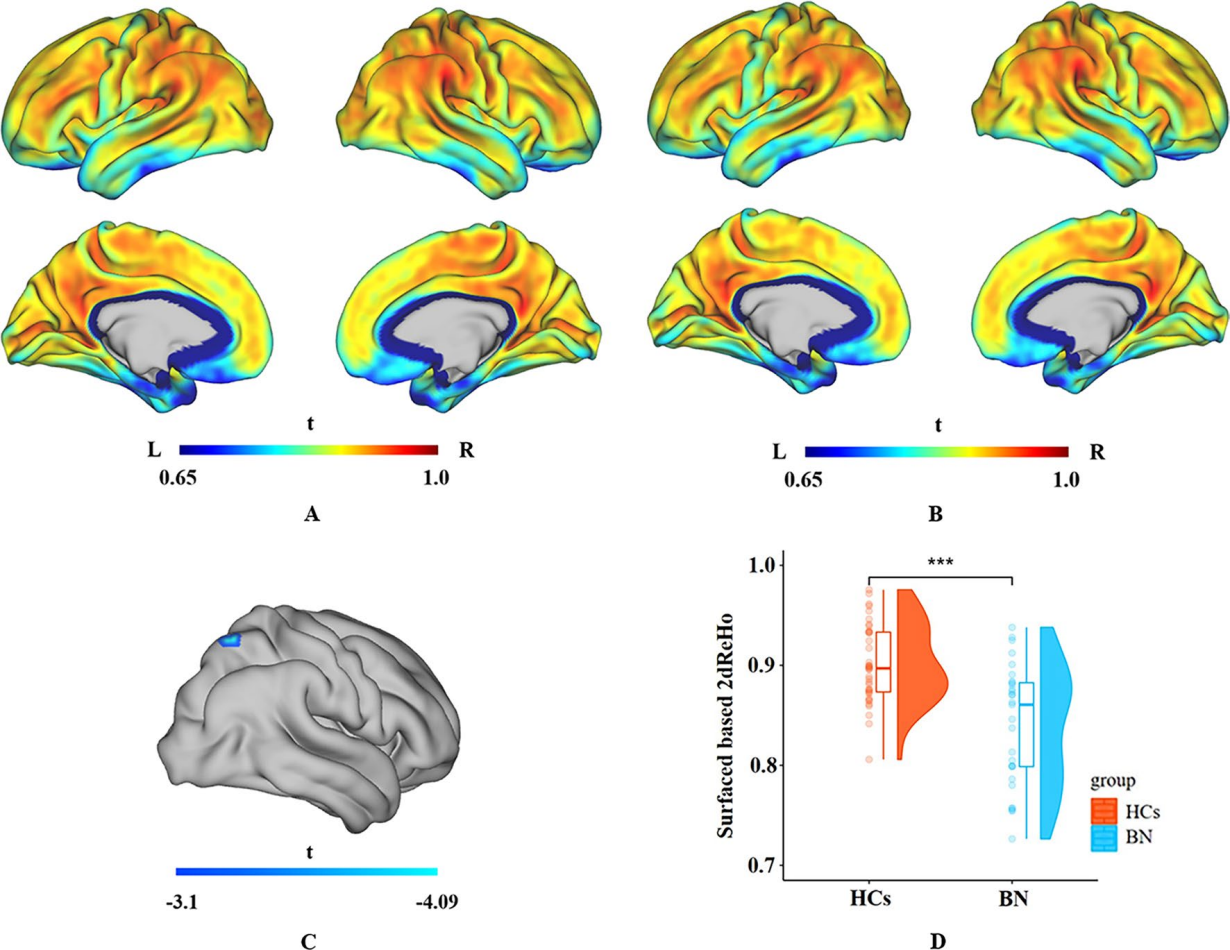
Average surface-based 2dReHo in HCs (**A**) and patients with BN (**B**) at the global level across the cortical surface. The cool-color area (**C**) indicates decreased surface-based 2dReho in the right SPL in patients with BN compared with HCs across the cortical surface (*p* < 0.05, random field theory corrected). Group differences in surface-based 2dReho in the right SPL are shown in the raincloud plots (**D**). *2dReho* two-dimensional regional homogeneity, *SPL* superior parietal lobule, *BN* bulimia nervosa, *HCs* healthy controls

Compared with the HCs, the patients with BN showed decreased surface-based 2dReho in the right superior parietal lobule (SPL) (*t* = − 4.09, *p* < 0.05) (Fig. 1; Table 2).

**Table 2:** Brain regions showing differences in the surface-based 2dReHo in patients with BN compared with HCs

Brain regions	Peak MNI coordinates			Cluster size	Peak *t* value
	*X*	*Y*	*Z*		
Right superior parietal lobule	24	− 60	67	17^a^	− 4.09

The cluster threshold is 17, vertex *p*<0.001, cluster *p*<0.05(RFT correction),* 2dReHo* two-dimensional regional homogeneity, *MNI* Montreal Neurological Institute, *BN* bulimia nervosa, *HCs* healthy controls

^a^Vertex size

### FC patterns in patients with BN

**Fig. 2:**
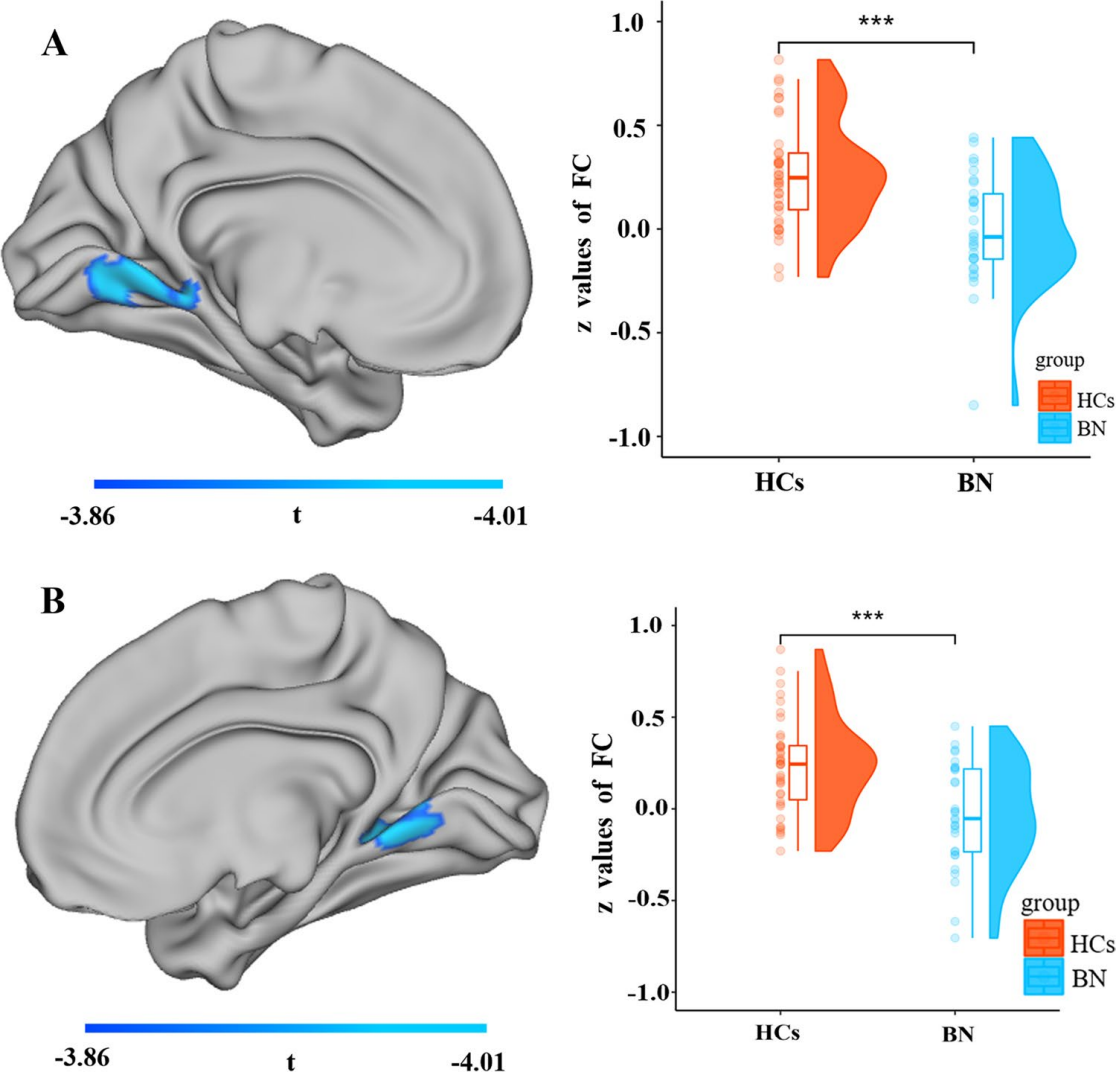
The cool-colored areas indicate that the left lingual gyrus (**A**) and right lingual gyrus (**B**) have decreased FC with the right SPL in people with BN compared to HCs. The raincloud plots indicate the group differences in *z* values of FC between the right SPL and the bilateral lingual gyrus. *FC* functional connectivity, *SPL* superior parietal lobule, *BN* bulimia nervosa, *HCs* healthy controls

**Fig. 3:**
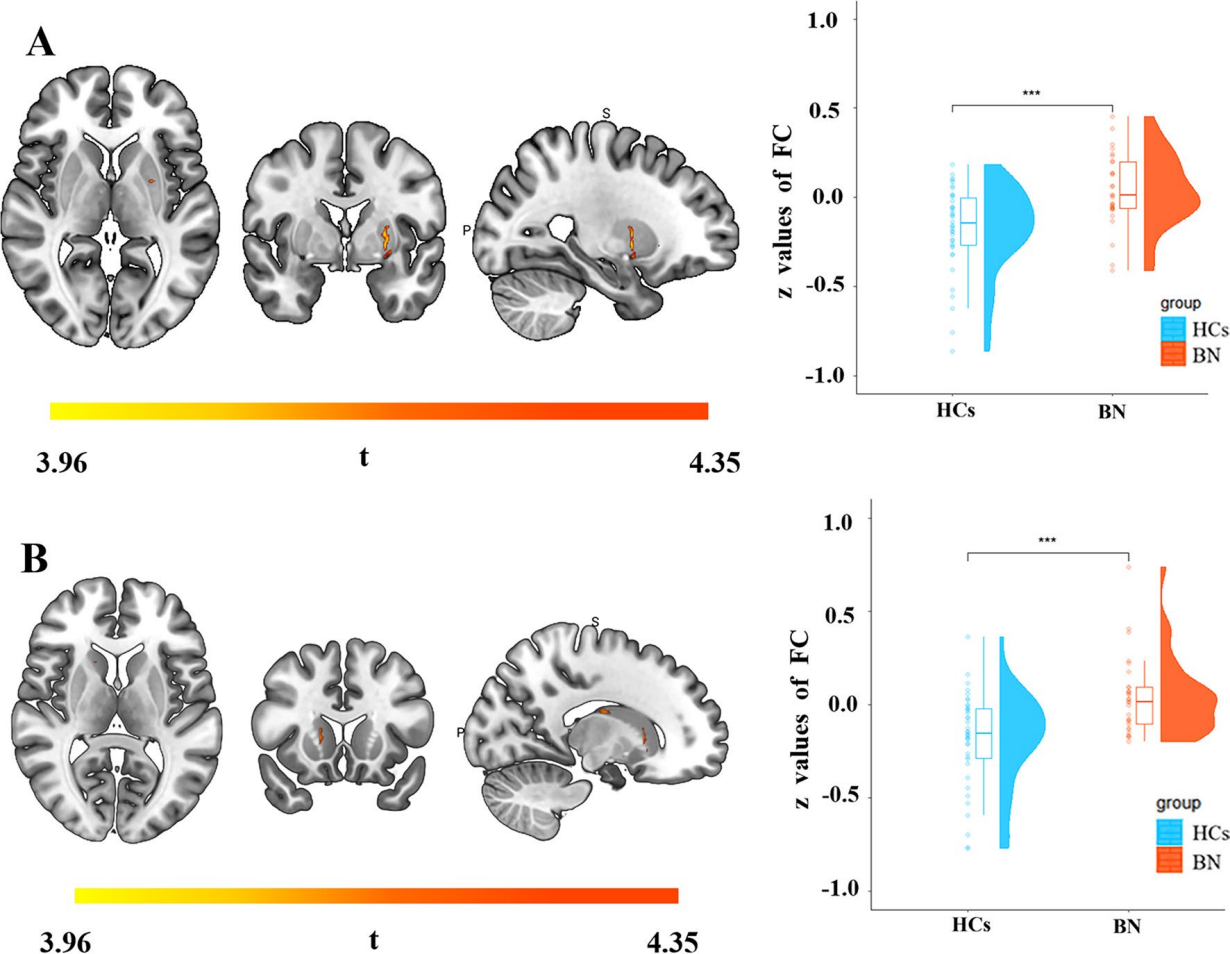
The warm-colored areas indicate that the right putamen (**A**) and left caudate (**B**) have increased FC with the right SPL in people with BN compared to HCs. The raincloud plots indicate the group differences in z values of FC between the right SPL and the right putamen and left caudate. *FC* functional connectivity, *SPL* superior parietal lobule, *BN* bulimia nervosa, *HCs* healthy controls

The right SPL, which showed significant changes in surface-based 2dReho, was selected as the seed region. Decreased FC was observed between the right SPL and the left lingual gyrus (*t* = − 3.86, *p* < 0.05) and right lingual gyrus (*t* = − 4.01, *p* < 0.05) (Fig. 2; Table 3). Increased FC was observed between the right SPL and the left caudate nucleus (*t* = 3.96, *p* < 0.05) and right putamen (*t* = 4.35, *p* < 0.05) (Fig. 3; Table 3).

**Table 3:** Alteration of functional connectivity between the right superior parietal lobule and other brain regions in BN patients compared with HCs

	Peak MNI coordinates				
Brain regions	*x*	*y*	*z*	Cluster size	Peak *t* value
Right lingual gyrus	14	− 58	− 9	143^a^	− 4.01
Left lingual gyrus	− 12	− 72	− 8	181^a^	− 3.86
Left caudate nucleus	− 18	− 10	24	107^b^	3.96
Right putamen	32	2	4	67^b^	4.35

In the t value, “–” represents the decreased functional connectivity between the right superior parietal lobule and brain regions in patients with BN compared with HCs

The cluster threshold is 143, vertex *p* < 0.001, cluster *p* < 0.05 (RFT correction for surface); FDR correction in voxel wise for subcortex; *MNI* Montreal Neurological Institute, *BN* bulimia nervosa, *HCs* healthy controls

^a^Vertex size

^b^Voxel size

To ensure the results were not influenced by male patients, we conducted a reanalysis of the data after excluding them. For further details, please refer to the supplementary material (Tables S2, S3).

### Correlations of clinical variables with FC and ReHo values

**Fig. 4:**
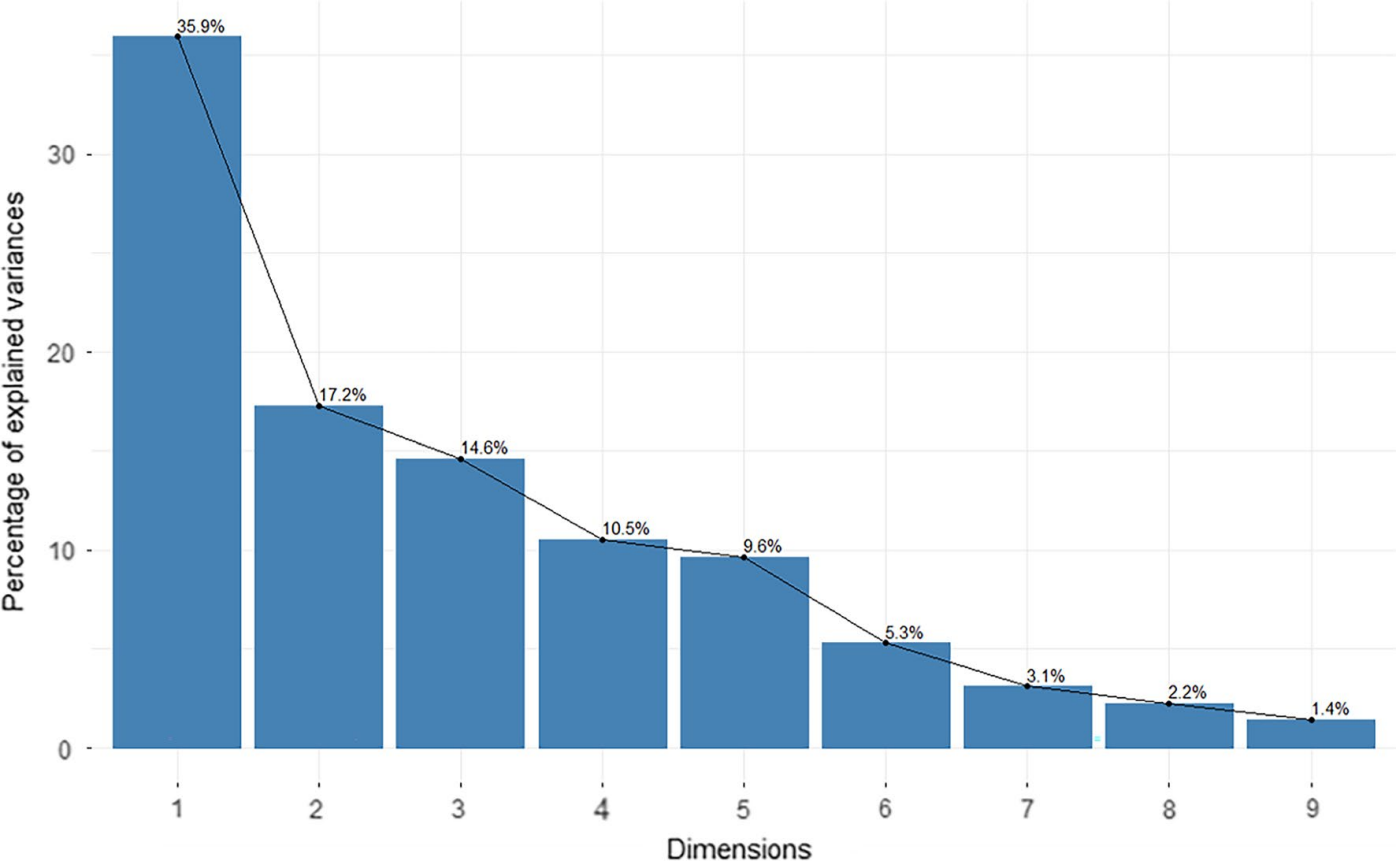
A scree plot for latent dimensions of behavioral traits identified in association with BN using PCA. The first three latent dimensions that were found to be significant accounted for 67.7% (PC1, 35.9%; PC2, 17.2%; PC3, 14.6%) of the variation among patients with BN. *BN* bulimia nervosa, *PCA* principal component analysis, *PC1* first principal component, *PC2* second principal component, *PC3* third principal component

**Fig. 5:**
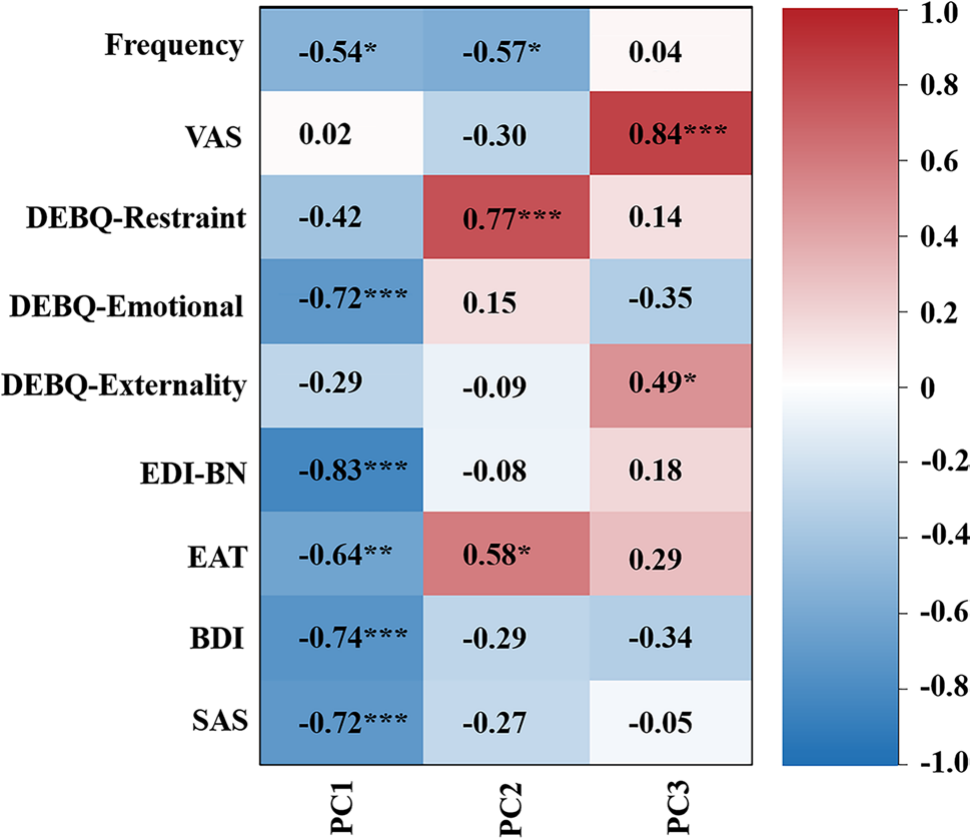
The *r* value matrix containing nine variables indicates the strength of correlation between the behavioral traits and the first three principal components. The correlation matrix was corrected by FDR (*P* < 0.05). *VAS* Visual Analog Scale, *Frequency* the number of binge-eating/purging episodes per week, *DEBQ* Dutch Eating Behavior Questionnaire, *EDI-BN* Eating Disorders Inventory-Bulimia Nervosa, *EAT* Eating Attitude Test, *BDI* Beck Depression Inventory, *SAS* Self-rating Anxiety Scale, *PC1* first principal component, *PC2* second principal component, *PC3* third principal component, *FDR* false discovery rate

The PCA identified the first three main latent dimensions (R^2^ values are 0.46, 0.21, and 0.18, respectively), accounting for 67.7% of the variation in the rank scales of behavioral traits among patients with BN (Fig. 4). The first principal component (PC1) was correlated with seven behavioral measures: the frequency of binge-eating/purging episodes per week and the DEBQ-Restraint, DEBQ-Emotional, EDI-BN, EAT, BDI and SAS scores (range *r* = − 0.42 to − 0.83). The second principal component (PC2) was correlated with the frequency of binge-eating/purging episodes per week (*r* = − 0.57) and the DEBQ-Restraint (*r* = 0.77) and EAT (*r* = 0.58) scores. The third principal component (PC3) was correlated with VAS (*r* = 0.84) and DEBQ-Externality (*r* = 0.49) scores (Fig. 5). (*P* < 0.05, FDR correction).

**Fig. 6:**
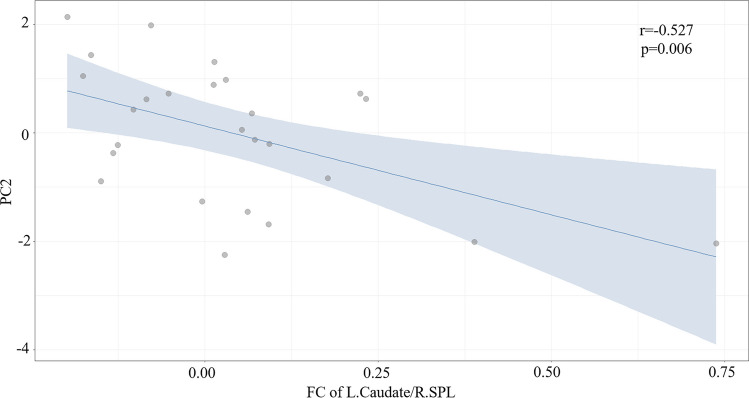
Negative correlations were found between PC2 and z values of FC between the right SPL and the left caudate nucleus (*r* = − 0.527, *p* = 0.006). (*p* < 0.05, FDR correction). *PC2* second principal component, *FC* functional connectivity, *R. SPL* right superior parietal lobule, *L. Caudate* left caudate nucleus, *FDR* false discovery rate

**Fig. 7:**
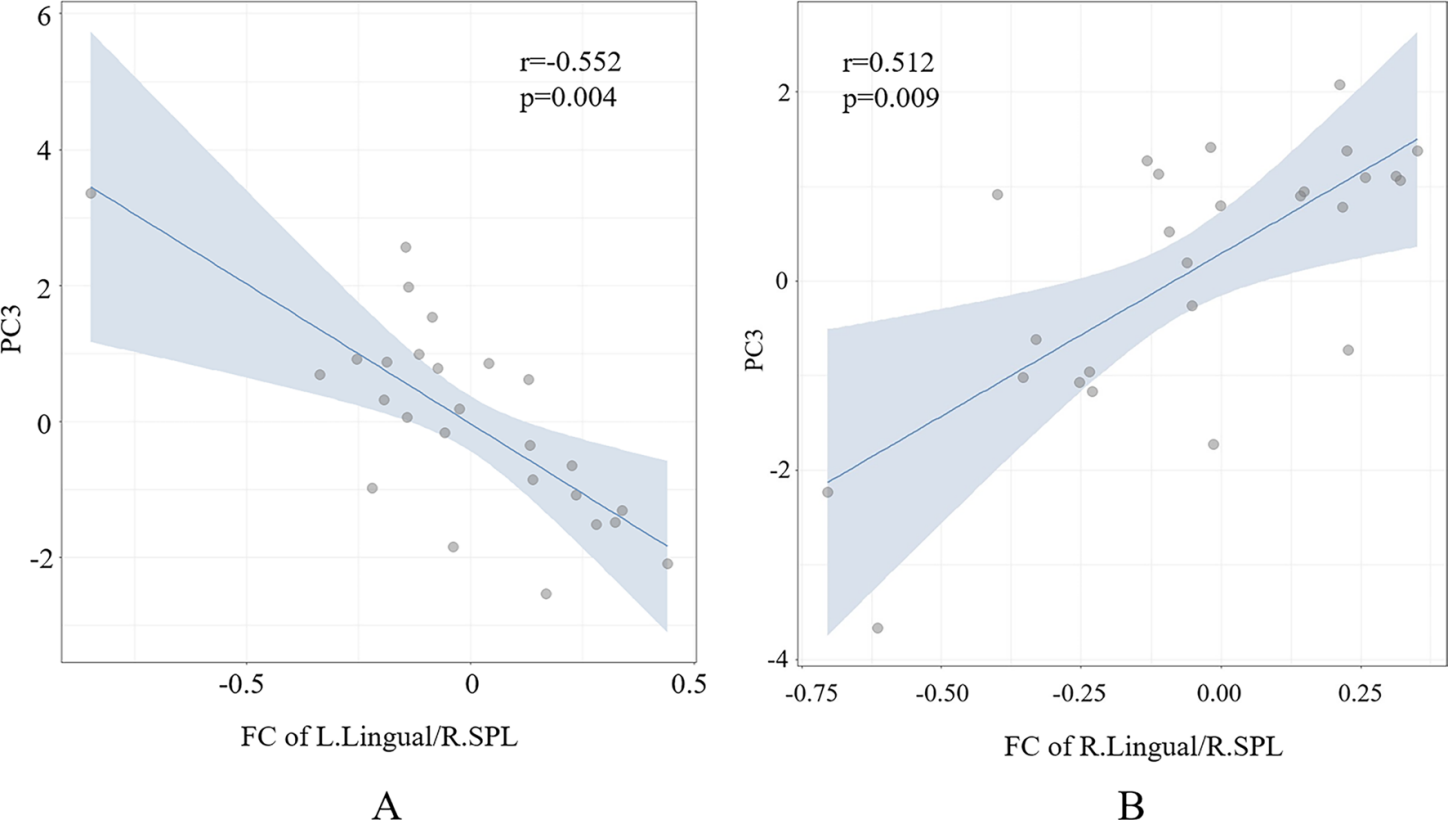
**A** Negative correlations were found between PC3 and *z* values of FC between the right SPL and the left lingual gyrus (*r* = − 0.552, *p* = 0.004). **B** Positive correlations were found between PC3 and *z* values of FC between the right SPL and the right lingual gyrus (*r* = 0.512, *p* = 0.009) (*p* < 0.05, FDR correction). *PC3* third principal component, *FC* functional connectivity, *R. SPL* right superior parietal lobule, *L. lingual* left lingual gyrus, *R. lingual* right lingual gyrus, *FDR* false discovery rate

In the FC-behavior association analysis, the general linear regression model of PC2-FC was reserved with *F* = 9.23 (*p* = 0.006) and adjusted *R*^2^ = 0.248 (Table 4), and the general linear regression model of PC3-FC was also reserved with *F* = 5.14 (*p* = 0.014) and adjusted *R*^2^ = 0.249 (Table 5). A significant main effect was found, as PC2 was negatively correlated with FC between the right SPL and the left caudate nucleus (*r* = − 0.527, *p* = 0.006) (Fig. 6). PC3 was negatively correlated with FC between the right SPL and the left lingual gyrus (*r* = − 0.552, *p* = 0.004) and positively correlated with FC between the right SPL and the right lingual gyrus (*r* = 0.512, *p* = 0.009) (Fig. 7). There was no significant main effect between PC1 and the alteration of FC in the BN group, please refer to the supplementary material (Table S4).

**Table 4:** The general linear regression model of PC2-FC

	β	Std.E	*t*	*p*	[95% CI of β]	*r* (partial)
FC of L.Caudate/R.SPL	− 0.527	(0.173)	− 3.038	0.006	[− 0.885, − 0.169]	− 0.527

*F* (1,27) = 9.23, *p* = 0.006, *R*^2^ = 0.278 (adjusted *R*^2^ = 0.248), *N* = 26 (BN patients)

*β* standardized coefficients, *Std.E* standard deviation error, *CI* confidence interval

**Table 5:** The general linear regression model of PC3-FC

	β	Std.E	*t*	*p*	[95% CI of β]	*r* (partial)
FC of L.Lingual/R.SPL	− 0.958	(0.301)	− 3.176	0.004	[− 1.581, − 0.334]	− 0.552
FC of R.Lingual/R.SPL	− 0.861	(0.301)	2.855	0.009	[0.237, 1.485]	0.512

*F* (2,26) = 5.14, *p* = 0.014, *R*^2^ = 0.309 (adjusted *R*^2^ = 0.249), *N* = 26 (BN patients)

β standardized coefficients, *Std.E* standard deviation error, *CI* confidence interval

In addition, in the results of the correlation analysis, we found the presence of some FC outliers. To ensure the reliability and validity of the results, we removed these FC outliers and recalculated the correlation between PCA components and FC. In the general linear regression model of PC2-FC, PC2 was still negatively correlated with FC between the right SPL and the left caudate nucleus (*r* = − 0.474, *p* = 0.017) when we removed one FC outlier (Table S5, Fig. S1). In the general linear regression model of PC3-FC, PC3 was still negatively correlated with FC between the right SPL and the left lingual gyrus (*r* = − 0.586, *p* = 0.005) and positively correlated with FC between the right SPL and the right lingual gyrus (*r* = 0.590, *p* = 0.005) when we removed two FC outliers (Table S6, Fig. S2).

## Discussion

In the present study, we performed surface-based 2dReho and seed-based FC analyses to investigate alterations in neural activity in patients with BN in the resting state. First, the present study identified the cortical activity of the right SPL as a key node through a surface-based 2dReHo analysis. Second, the FC results indicate that there were altered functional activities between the key nodes and multiple functional regions at the whole-brain level, which may be involved in vision, attention, memory, reward, and cognitive processes. These changes in neural activity have a correlation with maladaptive eating behavior in patients with BN. Our research may provide further useful information for the potential neuromodulation therapy of BN.

The SPL is an important region involved in a variety of functional roles, including visuospatial information integration, attention shifting, and memory [32]. Visual information processing occurs in two streams: the ventral and dorsal streams. Based on the anatomy and function of the dorsal stream, the dorso-dorsal and ventro-dorsal streams have been proposed, with the SPL serving as the termination site for dorso-dorsal streams [33]. In the dorsal stream, visuospatial information is processed for action control, indicating that the SPL, as a termination site of the dorso-dorsal stream, is associated with space perception [34].

Research by Wu et al. demonstrated that the right SPL plays a more dominant role in visuospatial attention compared to the left SPL in a visuomotor and visuospatial task-state fMRI study [35]. Additionally, task studies focusing on visual target attention shifts and spatially related attention shifts revealed bilateral activation of the SPL, suggesting that it may have a specific, transient function in shifting between different attentive states rather than maintaining a single specific attentive stat [36, 37]. Moreover, it has been suggested that the SPL may also play a functional role in the manipulation and rearrangement of memory for visual-spatial stimuli [38]. In the current study, alterations were observed in the regional cortical functional activity of the SPL in patients with BN compared to HCs. Based on findings from previous literature, these changes may involve alterations in visual-spatial information processing, attention, and memory in individuals with BN.

In addition to the functional role of the SPL in the memory processing of visuospatial stimuli, the lingual gyrus has been consistently implicated in visual memory and the encoding of visual information based on the presented stimuli [39]. Previous studies using dietary recall methods have demonstrated that individuals who engage in binge eating tend to overestimate the amount of food consumed, suggesting that their reliance on memory could lead to imprecise evaluation [40, 41]. Building upon these previous findings and our own results, the altered neural activity in memory-related brain regions in patients with BN may contribute to incorrect evaluation of food intake, leading to maladaptive eating behavior. However, further confirmation of the link between neural activity in memory-related brain regions and maladaptive eating behaviors in BN, such as intermittent binge eating, requires investigation using task-based designs.

Moreover, other studies have highlighted the functional activation of the lingual gyrus in relation to the perception of pain, happiness, and loneliness, suggesting a possible role of this cortical region in specific emotional processes [42, 43]. Zhang et al. also found that depressed individuals exhibit significantly lower nodal centralities in the lingual gyrus in small-world functional brain networks [44]. These findings may be relevant to anxiety, depression, and emotional eating (eating in response to emotional distress) in patients with BN [45]. The results of our study also indicated that individuals with BN scored significantly higher than HCs on the SAS, BDI, and DEBQ-Emotional scale. PC3, which primarily reflects the externality of eating behaviors in patients with BN, exhibited a negative correlation with FC between the right SPL and the left lingual gyrus, and a positive correlation with FC between the right SPL and the right lingual gyrus. These correlations suggest potential alterations in visuospatial modulation and reorganization of visual cortex activity among patients with BN. Furthermore, these changes in FC may also play a role in altered food cravings experienced by individuals with BN when they encounter food stimuli, thereby contributing to the external eating behavior observed in BN patients. Additionally, PC3 showed a correlation with VAS scores in patients with BN, suggesting a potential influence of hunger/satiety on functional brain activity. We aim to further confirm this relationship in future studies using a task-state design in patients with BN.

The caudate nucleus and putamen, which constitute the dorsal striatum, not only represent the largest component of the basal ganglia but also play a crucial role in the mesocorticolimbic reward pathways [46]. Apart from their involvement in reward-related processes, these regions also support cognitive functions [47, 48]. In a study by Wang et al. [9], altered whole-brain FC of striatal subregions, including the caudate nucleus and putamen, was observed in BN. Our own findings of altered FC between the SPL and the caudate nucleus and putamen may suggest that patients with BN exhibit biased evaluation of food reward value. Additionally, these results may indicate some extent of alteration in food-related cognitive function among individuals with BN.

The PC2, mainly associated with the number of binge-eating/purging episodes and restrictive eating behavior, showed a correlation with the FC between the SPL and the caudate nucleus. The potential explanation is that the altered FC between the SPL and caudate nucleus could bring about changes in visual, reward processing, and cognitive functions, subsequently resulting in maladaptive eating behaviors (such as binge-eating/purging episodes and restrictive eating behaviors). To delve deeper into the specific functions implicated in the altered FC between the SPL and the observed striatum regions, further investigations using various task designs, such as food/money reward paradigms and cognitive control tasks (e.g., the Stroop test and cued task switching), are warranted. While PC1 was not associated with FC between the SPL and other brain regions, it accounted for approximately one-third of the variance. Given that PC1 is associated with seven behavioral measures, one could hypothesize that this principal component primarily reflects the individual's subjective emotions, including external eating, emotional eating, anxiety, and depression.

This work still some limitations that should be addressed in future studies. First, although the present study controlled the resting-state condition of all participants during the scan, it has been found that the resting-state condition of participants (eyes closed, eyes open, or eyes open fixated) during the fMRI scan has effects on functional network connectivity, such as the connectivity of auditory and sensorimotor regions [49]. Therefore, in future neuroimaging studies related to eating disorders, we will more strictly control the scanning conditions of the participants. Second, FC was defined as the correlation or covariance of the properties of activity in different brain regions, which is a type of functional correlation. This approach may not necessarily reflect the true FC of brain regions, but it is the most efficient and most commonly used approach. One should be careful not to overstate the implications of FC results when interpreting them. Third, the phase of the menstrual cycle has been shown to affect the neural activation associated with reward [50–53]. Therefore, we will take into account the possible effects of women's menstrual cycles on the results in a future experimental design. Fourth, although we assessed the participants' handedness through verbal inquiries (e.g., their preferred hand for activities such as writing, drawing, throwing, brushing teeth, or using a spoon), and identified all participants as right-handed, we acknowledge that we did not use a comprehensive measure of cerebral lateralization, such as the Edinburgh Handedness Inventory. In future research, we plan to implement more detailed scales to accurately assess the handedness of the participants.

## Conclusions

In conclusion, this study reveals that patients with BN have altered surfaced-based neural activity and FC in resting-state fMRI. We found that in BN, the right SPL, as a key node, exhibits reorganization of FC with multiple brain regions, including regions related to vision, reward, and cognitive processes. The FC between the right SPL and the lingual gyrus, which is located in the visual pathway, is weakened, which may reflect altered visuospatial modulation. The alteration of FC between the right SPL and reward-related regions, including the caudate nucleus and putamen, may be related to changes in the evaluation of food reward. Furthermore, brain reorganization may be correlated with maladaptive eating behaviors. These findings provide useful information to understand the neural mechanisms of BN in depth, and they also identify new potential targets for the neuromodulatory treatment of BN.

### Strength and limits

- First to study the resting-state surface-based neural activity in patients with BN
- We found right SPL, as a key node, exhibits reorganization of FC with multiple brain regions, which may be correlated with maladaptive eating behaviors
- These findings provide useful information to understand the neural mechanisms of BN and new potential targets for the neuromodulatory treatment of BN
- The resting-state condition of participants during the fMRI scan has effects on functional network connectivity, we will more strictly control the scanning conditions of the participants
- The phase of the menstrual cycle has been shown to affect the neural activation associated with reward. Therefore, we will take into account the possible effects of women's menstrual cycles on the results in a future experimental design.

### What is already known on this subject?

In recent years, major advances in neuroimaging in the field of neuroscience, including improvements in functional magnetic resonance imaging (fMRI), have increased the knowledge of the interrelationship between neural mechanisms and BN. However, little is known about the alterations in cerebral cortex activity in patients with BN, and the relationship between cerebral cortex activity and maladaptive eating behaviors.​

### What this study adds?

This study reveals that patients with BN have altered surfaced-based neural activity and FC in resting-state fMRI. We found that in BN, the right SPL, as a key node, exhibits reorganization of FC with multiple brain regions, including regions related to vision, reward, and cognitive processes. In addition, reorganization of neural activity in the cerebral cortex may be associated with maladaptive eating behaviors. These findings provide useful information to understand the neural mechanisms of BN in depth, and they also identify new potential targets for the neuromodulatory treatment of BN.

### Supplementary Information

Below is the link to the electronic supplementary material.

Supplementary file1 (TIF 1232 KB)

Supplementary file2 (TIF 2172 KB)

Supplementary file3 (DOCX 72 KB)

## Data Availability

The data that support the findings of this study are available from the corresponding author upon reasonable request.
