# Supplementary figures and images for: Uncovering neural pathways underlying bulimia nervosa: resting-state neural connectivity disruptions correlate with maladaptive eating behaviors
Source: Eat Weight Disord. 2023 Oct 30;28(1):91. doi: 10.1007/s40519-023-01617-5 (PMC10613592; doi:10.1007/s40519-023-01617-5)

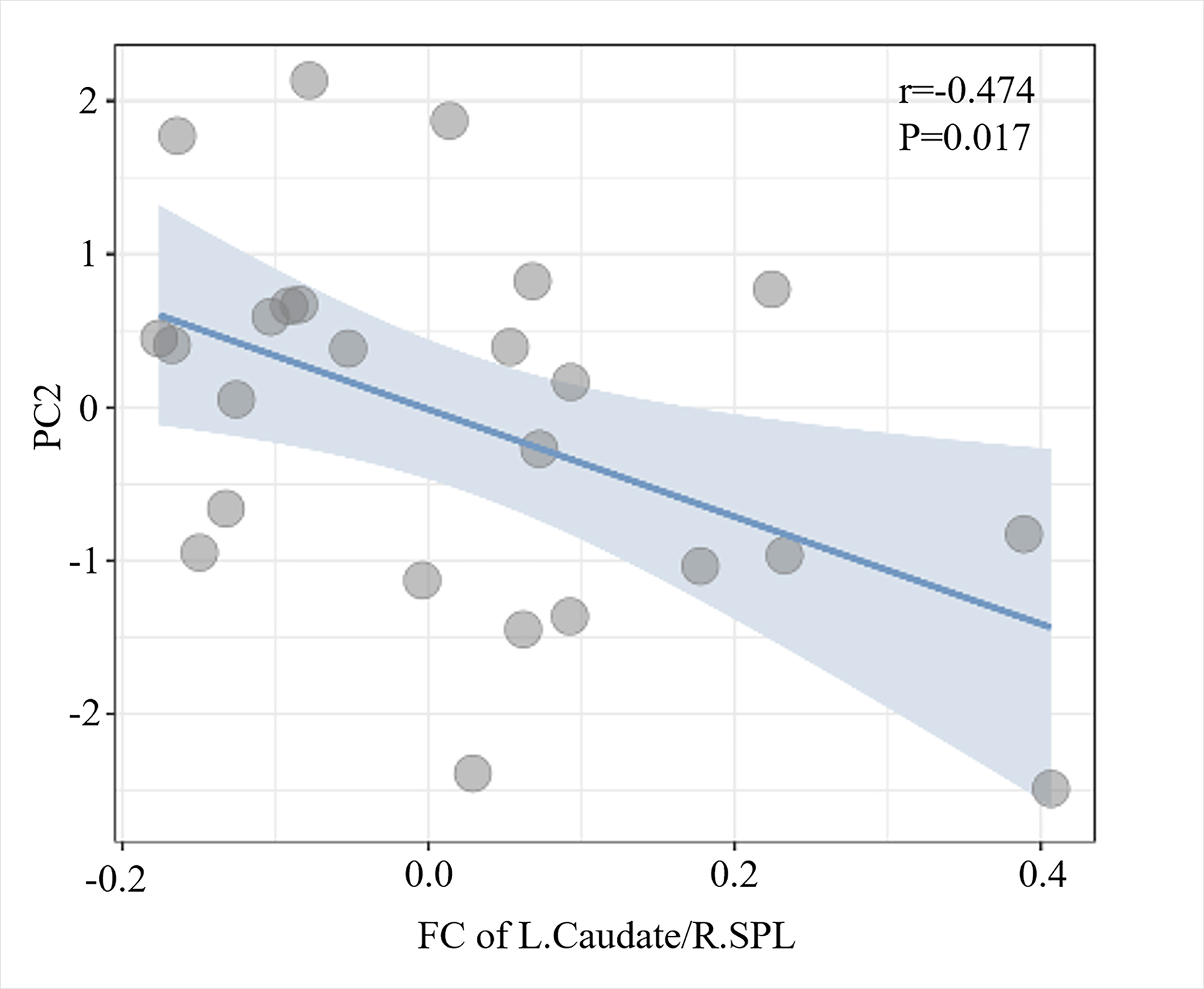

Supplement: Supplementary file 1 — Supplementary file1 (TIF 1232 KB) [file 40519_2023_1617_MOESM1_ESM.tif]

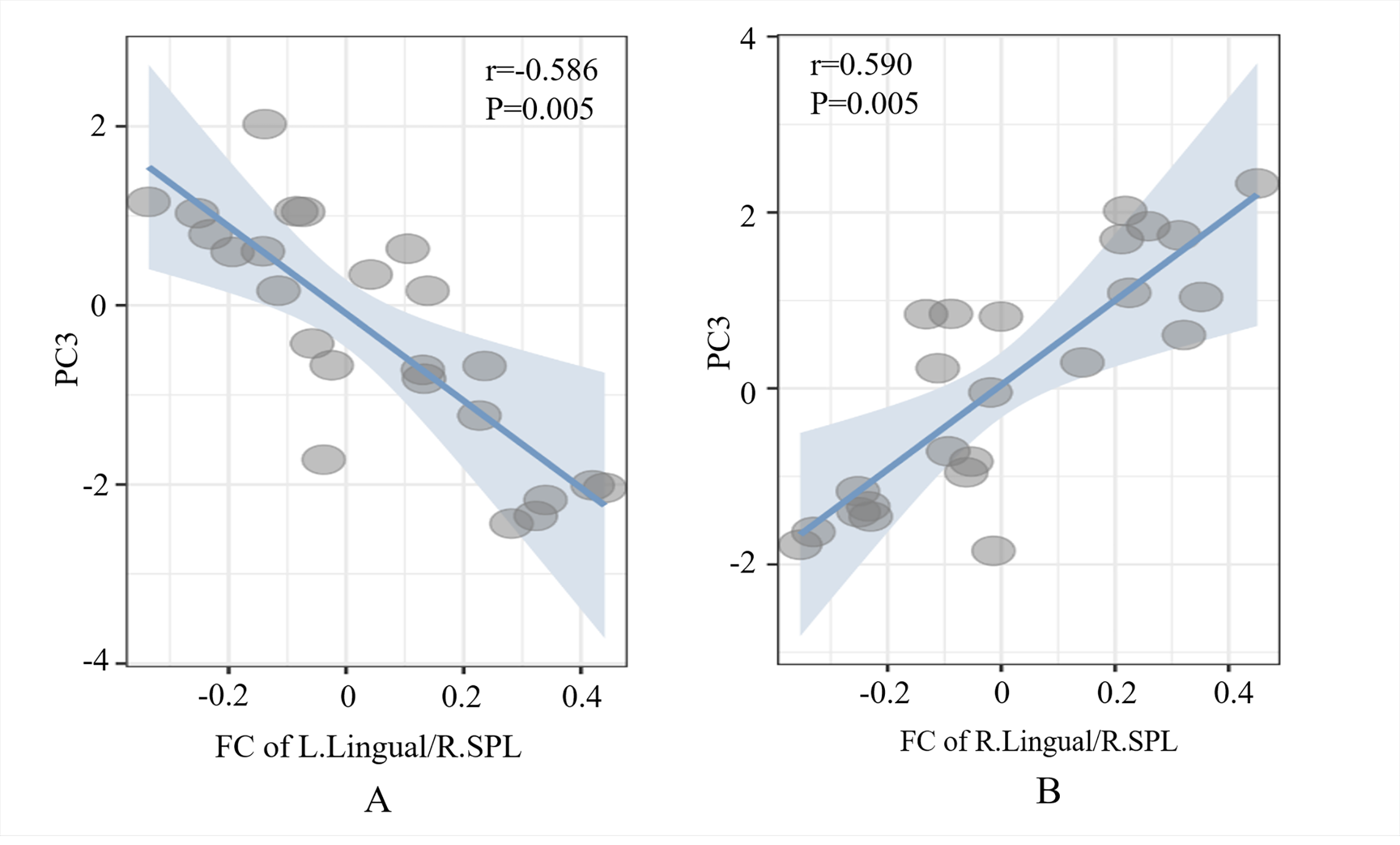

Supplement: Supplementary file 2 — Supplementary file2 (TIF 2172 KB) [file 40519_2023_1617_MOESM2_ESM.tif]
