# Supplementary material for: Uncovering neural pathways underlying bulimia nervosa: resting-state neural connectivity disruptions correlate with maladaptive eating behaviors
Source: Eat Weight Disord. 2023 Oct 30;28(1):91. doi: 10.1007/s40519-023-01617-5 (PMC10613592; doi:10.1007/s40519-023-01617-5)
Supplement: Supplementary file 3 — Supplementary file3 (DOCX 72 KB) [file 40519_2023_1617_MOESM3_ESM.docx]

**Preprocessing of fMRI data**

First, a reference volume and its skull-stripped version were generated using a custom methodology of fMRIprep. A deformation field to correct for susceptibility distortions was estimated based on fMRIPrep’s fieldmap-less approach. The deformation field was that resulting from coregistering the BOLD reference to the same-participant T1w reference with its inverted intensity[1, 2]. Registration was performed with antsRegistration (ANTs 2.3.3), and the process was standardized by constraining deformation to be nonzero only along the phase-encoding direction and modulated with an average fieldmap template[3]. Based on the estimated susceptibility distortion, a corrected EPI reference was calculated for a more accurate coregistration with the anatomical reference. The BOLD reference was then coregistered to the T1w reference using bbregister (FreeSurfer), which implements boundary-based registration[4]. Coregistration was configured with six degrees of freedom. Head-motion parameters with respect to the BOLD reference (transformation matrices and six corresponding rotation and translation parameters) were estimated before any spatiotemporal filtering using mcflirt (FSL 5.0.9)[5]. BOLD runs were slice-time corrected using 3dTshift from AFNI 20160207 (RRID:SCR_005927)[6]. The BOLD time series were then resampled onto the following surfaces (FreeSurfer reconstruction nomenclature): fsaverage by using mri_vol2surf (FreeSurfer). Then, grayordinates files[7] containing 91k samples for each participant were also generated using the highest-resolution fsaverage as an intermediate standardized surface space.

Several confounding time series were calculated based on the preprocessed BOLD: FD and three regionwise global signals. FD was computed following Power (absolute sum of relative motions)[8]. The three global signals were extracted within the CSF, WM, and whole-brain masks. Outputs from fMRIPrep[9] were postprocessed using the XCP-D[10, 11]. For each of the 1 CIFTI runs per participant, the following postprocessing was performed: before nuisance regression and filtering, any volumes with FD values greater than 0.3 were flagged as outliers and excluded from nuisance regression[8, 10, 12]. In total, 36 nuisance regressors were selected from the nuisance confound matrices of the fMRIPrep output. These nuisance regressors included six motion parameters; the global signal, the mean WM signal, and the mean CSF signal with their temporal derivatives; the quadratic expansion of the six motion parameters; and the tissues signals and their derivatives[10, 11]. These nuisance regressors were regressed from the bold data using linear regression as implemented in Scikit-Learn 0.23.1[13]. Residual time series from this regression were then bandpass filtered within the frequency band 0.01-0.08 Hz.

**Table S1. ​The number of patients with bulimia nervosa included/excluded according to comorbidity category**

Anxiety Depression Bipolar Disorder Schizophrenia

No. 10 19 2 2

No., the number of patients with BN. In the present study, 10 patients with BN had comorbid anxiety, and 19 had comorbid depression. We excluded 4 patients with BN because 2 patients had comorbid bipolar disorder and 2 had comorbid schizophrenia.

**Table S2. Brain regions showing differences in the surface-based 2dReHo between female patients with BN and HCs**

Peak MNI coordinates

Brain regions X Y Z Cluster size Peak t value

Right superior parietal lobule 23 -62 66 14^a^ -3.96

The cluster threshold is 14, vertex *p* <0.001, cluster *p* <0.05 (RFT correction).

2dReHo, two-dimensional regional homogeneity; MNI, Montreal Neurological Institute; BN, bulimia nervosa; HCs, healthy controls.

^a^ vertex size.

**Table S3. Alteration in functional connectivity between the right superior parietal lobule and other brain regions in female patients with BN compared to HCs.**

Peak MNI coordinates

Brain regions x y z Cluster size peak t value

Right lingual gyrus 14 -59 -5 156^a^ -3.92

Left lingual gyrus -14 -69 -8 192^a^ -3.89

Left caudate nucleus -16 -9 26 91^b^ 3.99

Right putamen 31 3 6 43^b^ 4.20

“-” represents the decreased functional connectivity between the right superior parietal lobule and brain regions in patients with BN compared with HCs.

The cluster threshold is 156, vertex *p* <0.001, cluster *p* <0.05(RFT correction for surface); FDR correction in voxel wise for subcortex; MNI, Montreal Neurological Institute; BN, bulimia nervosa; HCs, healthy controls.

^a^ vertex size.

^b^ voxel size.

**Table S4. The general linear regression model of PC1-FC**

β Std.E t *p* [95% CI of β] r(partial)

FC of R.putamen/R.SPL 0.263 (0.192) 1.367 0.186 [-0.137, 0.662] 0.286

FC of L.Caudate/R.SPL -0.436 (0.227) -1.917 0.069 [-0.909, 0.037] -0.386

FC of L.Lingual/R.SPL 0.527 (0.329) 1.603 0.124 [-0.157, 1.210] 0.330

FC of R.Lingual/R.SPL -0.743 (0.325) -2.288 0.033 [-1.418, -0.068] -0.447

F (4,24) =2.20, *p*=0.104

R^2^= 0.309 (Adjusted R^2^=0.249)

N= 26 (BN patients)

β, standardized coefficients; Std.E, Standard Deviation Error; CI, confidence interval

**Table S5. The general linear regression model of PC2-FC**

β Std.E t *p* [95% CI of β] r(partial)

FC of L.Caudate/R.SPL -0.474 (0.184) -2.583 0.017 [-0.854, -0.094] -0.474

F (1,23) =6.67, *p*=0.017

R^2^= 0.225 (Adjusted R^2^=0.191)

N= 25 (BN patients)

β, standardized coefficients; Std.E, Standard Deviation Error; CI, confidence interval

**Table S6. The general linear regression model of PC3-FC**

β Std.E t *p* [95% CI of β] r(partial)

FC of L.Lingual/R.SPL -1.126 (0.357) -3.150 0.005 [-1.874, -0.378] -0.586

FC of R.Lingual/R.SPL 1.183 (0.372) 3.182 0.005 [0.405, 1.960] 0.590

F (4,19) =3.23, *p*=0.035

R^2^= 0.405 (Adjusted R^2^=0.280)

N= 24 (BN patients)

β, standardized coefficients; Std.E, Standard Deviation Error; CI, confidence interval

**Fig.S1.** Negative correlations were found between PC2 and z values of FC between the right SPL and the left caudate nucleus (r=-0.474, *P*=0.017). (*P* < 0.05, FDR correction). PC2, second principal component; FC, functional connectivity; R. SPL, right superior parietal lobule; L. Caudate, left caudate nucleus; FDR, false discovery rate.

**Fig.S2.** A. Negative correlations were found between PC3 and z values of FC between the right SPL and the left lingual gyrus (r=-0.586, *P*=0.005). B. Positive correlations were found between PC3 and z values of FC between the right SPL and the right lingual gyrus (r=0.590, *P*=0.005). (*P* < 0.05, FDR correction). PC3, third principal component; FC, functional connectivity; R. SPL, right superior parietal lobule; L. Lingual, left lingual gyrus; R. Lingual, right lingual gyrus; FDR, false discovery rate.

**REFERENCES**

1. Huntenburg J., Gorgolewski K., Anwander A., Margulies D.: Evaluating nonlinear coregistration of BOLD EPI and T1 images, 2014.

2. Wang S., Peterson D.J., Gatenby J.C., Li W., Grabowski T.J., Madhyastha T.M.: Evaluation of Field Map and Nonlinear Registration Methods for Correction of Susceptibility Artifacts in Diffusion MRI. Front Neuroinform, 11, 17, 2017.<https://doi.org/10.3389/fninf.2017.00017>

3. Treiber J.M., White N.S., Steed T.C., Bartsch H., Holland D., Farid N., McDonald C.R., Carter B.S., Dale A.M., Chen C.C.: Characterization and Correction of Geometric Distortions in 814 Diffusion Weighted Images. PLoS One, 11, e0152472, 2016.<https://doi.org/10.1371/journal.pone.0152472>

4. Greve D.N., Fischl B.: Accurate and robust brain image alignment using boundary-based registration. NeuroImage, 48, 63-72, 2009.<https://doi.org/10.1016/j.neuroimage.2009.06.060>

5. Jenkinson M., Bannister P., Brady M., Smith S.: Improved optimization for the robust and accurate linear registration and motion correction of brain images. NeuroImage, 17, 825-841, 2002.<https://doi.org/10.1016/s1053-8119(02)91132-8>

6. Cox R.W., Hyde J.S.: Software tools for analysis and visualization of fMRI data. NMR in biomedicine, 10, 171-178, 1997.<https://doi.org/10.1002/(sici)1099-1492(199706/08)10:4/5><171::aid-nbm453>3.0.co;2-l

7. Glasser M.F., Sotiropoulos S.N., Wilson J.A., Coalson T.S., Fischl B., Andersson J.L., Xu J., Jbabdi S., Webster M., Polimeni J.R., Van Essen D.C., Jenkinson M., Consortium W.U.-M.H.: The minimal preprocessing pipelines for the Human Connectome Project. NeuroImage, 80, 105-124, 2013.<https://doi.org/10.1016/j.neuroimage.2013.04.127>

8. Power J.D., Mitra A., Laumann T.O., Snyder A.Z., Schlaggar B.L., Petersen S.E.: Methods to detect, characterize, and remove motion artifact in resting state fMRI. NeuroImage, 84, 320-341, 2014.<https://doi.org/10.1016/j.neuroimage.2013.08.048>

9. Esteban O., Markiewicz C.J., Blair R.W., Moodie C.A., Isik A.I., Erramuzpe A., Kent J.D., Goncalves M., DuPre E., Snyder M., Oya H., Ghosh S.S., Wright J., Durnez J., Poldrack R.A., Gorgolewski K.J.: fMRIPrep: a robust preprocessing pipeline for functional MRI. Nat Methods, 16, 111-116, 2019.<https://doi.org/10.1038/s41592-018-0235-4>

10. Satterthwaite T.D., Elliott M.A., Gerraty R.T., Ruparel K., Loughead J., Calkins M.E., Eickhoff S.B., Hakonarson H., Gur R.C., Gur R.E., Wolf D.H.: An improved framework for confound regression and filtering for control of motion artifact in the preprocessing of resting-state functional connectivity data. NeuroImage, 64, 240-256, 2013.<https://doi.org/10.1016/j.neuroimage.2012.08.052>

11. Ciric R., Rosen A.F.G., Erus G., Cieslak M., Adebimpe A., Cook P.A., Bassett D.S., Davatzikos C., Wolf D.H., Satterthwaite T.D.: Mitigating head motion artifact in functional connectivity MRI. Nat Protoc, 13, 2801-2826, 2018.<https://doi.org/10.1038/s41596-018-0065-y>

12. Satterthwaite T.D., Wolf D.H., Ruparel K., Erus G., Elliott M.A., Eickhoff S.B., Gennatas E.D., Jackson C., Prabhakaran K., Smith A., Hakonarson H., Verma R., Davatzikos C., Gur R.E., Gur R.C.: Heterogeneous impact of motion on fundamental patterns of developmental changes in functional connectivity during youth. NeuroImage, 83, 45-57, 2013.<https://doi.org/10.1016/j.neuroimage.2013.06.045>

13. Pedregosa F., Varoquaux G., Gramfort A., Michel V., Thirion B., Grisel O., Blondel M., Prettenhofer P., Weiss R., Dubourg V., Vanderplas J., Passos A., Cournapeau D., Brucher M., Perrot M., Duchesnay É.: Scikit-learn: Machine Learning in Python. J. Mach. Learn. Res., 12, 2825–2830, 2011
